# Pathological characteristics predicting sentinel lymph node metastasis in early breast cancer patients

**DOI:** 10.22088/cjim.15.3.472

**Published:** 2024-08-01

**Authors:** Mohammad Esmaeil Akbari, Atieh Akbari, Saba Ebrahimian

**Affiliations:** 1 Cancer Research Center, Shahid Beheshti University of Medical Sciences, Tehran, Iran; 2 Department of Surgery, Babol University of Medical Sciences, Babol, Iran

**Keywords:** Sentinle lymph node. Pathology. Metastasis .breast cancer

## Abstract

**Background:**

In this study, we aimed to identify the predicting pathological factors affecting sentinel lymph node biopsy (SLNB) in patients with clinically node-negative breast cancer.

**Methods:**

Our single institution retrospective study was conducted at the Cancer Research Center of Shahid Beheshti University of Medical Sciences from 2018 to 2021. Data were imported into and analyzed using SPSS Version 28 for Windows (IBM Corp., Armonk, NY, USA).

**Results:**

Of the 76 patients who underwent SLNB, 43 (56.6%) had negative SLNB and 33 (43.4%) had positive SLNB which led to axillary lymph node dissection (ALND). The relationship between hormone receptor status (ER/PR/Her2), pathology type (IDC, ILC, DCIS, LCIS), tumor size, and Ki67 expression was assessed. According to the results, axillary lymph node involvement can be predicted based on the scores and results of the three variables: IDC tumor type, lympho vascular invasion (LVI), and Ki67 expression. The positive relationship between IDC tumor type and LVI with SLNB indicates that with positive IDC tumor type and LVI, there is a higher probability of positive axillary lymph nodes (3.88 times higher probability for IDC tumor type and 6.75 times higher probability for the LVI factor). However, when the Ki67 expression is lower, the probability of positive axillary lymph nodes is higher (3.58 times higher probability).

**Conclusion:**

IDC tumor type, LVI, and lower Ki67 expression are independent predictive factors of positive SLNB.

One of the predictors of breast cancer survival is the involvement of axillary lymph nodes (1). The condition of the lymph nodes helps to predict the risk of local recurrence and distant metastasis and, as a result, to choose the appropriate adjuvant treatment for the patient (2). 

Axillary lymph node dissection (ALND) is a standard method for the dissection of axillary lymph nodes with clear boundaries. Despite a detailed knowledge of the technique and anatomy and the benefits of axillary lymph node dissection, complications such as pain, numbness, and lymphedema may occur afterward (3-6). Axillary lymph node dissection (ALND) has been and still is an important part of breast cancer treatment, although sentinel lymph node biopsy (SLNB) has revolutionized breast cancer surgery. SLNB provides the same information as ALND but with fewer complications. The results of several studies conducted in the past years have introduced SLNB as the gold standard for early breast cancer treatment (7). Sentinel lymph node biopsy is a modern technique used to assess the axillary stage in breast cancer patients. The fundamental principle underlying this approach is that by detecting and extracting a "sentinel node," information regarding the involvement of the entire lymph node can be obtained (8).

Today, SLNB is routinely used for breast cancer patients who do not have lymph node involvement in the clinical and radiologic before surgery evaluation. However, it requires facilities such as radiopharmaceutical injection and gamma camera equipment, and the cooperation of different departments, which in developing countries imposes a financial burden on treatment departments and may not always be available (6, 9, 10). Considering these limitations, if we could identify the predictors of positive sentinel lymph nodes, it may help to define a more precise indication for SLNB. The aim of this study was to identify all positive SLNBs in our patients with clinically node-negative breast cancer and to study the association between positive SLNB and patients' histopathological characteristics.

## Methods

Patients who were diagnosed with primary invasive breast cancer between 2018 and 2021 from the Cancer Research Center database at Shahid Beheshti University of Medical Science, with negative pre-surgery evaluations for axillary lymph nodes, and who underwent sentinel lymph node biopsy (SLNB) with the same surgical team, were included in the study. Data were retrospectively collected from the patients' electronic medical records. Patients who were treated outside the Cancer Research Center of Shahid Beheshti University of Medical Sciences Hospital, had not undergone SLNB, and patients with missing data were excluded. The pathology report of their axillary after surgery was analyzed.

Data were imported into and analyzed using SPSS Version 28 for Windows (IBM Corp., Armonk, NY, USA). Descriptive statistics (for continuous variables) and frequency tables (for categorical variables) including frequency percentage, mean, and standard deviation were used to describe the variables and summarize the predictors of positive SLNB. The relationship of variables and involved axillary lymph nodes was tested with Fisher's exact tests (for 2*2 relationships) (chi-square) and t-tests for independent groups (Ki67). Finally, a logistic binary regression test was used to predict the involvement of the axillary lymph nodes.

## Results

Out of 76 patients who underwent SLNB, 43 (56.6%) had no axillary lymph node involvement and the result was negative lymph node, while 33 (43.4%) had involvement of axillary lymph nodes, and a positive SLNB led to ALND. Table 1 describes predictor variables according to negative and positive SLNB. The significance of the relationship between predictor variables and SLNB involvement was checked with a chi-square test and Fisher's exact test (for two-valued variables). 

The results of table 1 showed that, with a 95% confidence level, a significant relationship was observed between invasive ductal carcinoma (IDC) tumor type and Lymphovascular invasion (LVI) and involvement of axillary lymph nodes (p < 0.05). Examining the frequency percentages demonstrated that patients with IDC tumor type had a higher percentage of positive SLNB. According to the results, 87.9% of patients with IDC tumor type had a positive SLNB, but in patients with other tumor types, this percentage was 65.1%.

Also, the findings showed that positive LVI had a statistically significant relationship with positive SLNB, and a higher percentage of positive SLNB was observed with positive LVI. The results demonstrated that 48.4% of patients with positive LVI had positive SLNB, but in those with negative LVI, positive SLNB was 12.2%.

**Table 1:** Description of predictive variables according to the status of axillary lymph node involvement along with chi-square and Fisher's exact test

**Variables**	**Levels**	**Negative SLNB**		**Positive SLNB**		**p**
		**Number**	**Percent**	**Number**	**Percent**	
**Tumor Size** **Centimeter**	T<=2	10	29.4	5	17.9	559/0
	2<T<=5	16	47.1	16	57.1	
	5<T	8	23.5	7	25	
**HER2**	Negative	15	45.5	10	35.7	602/0
	Positive	18	54.5	18	64.3	
**ER - PR**	Negative	11	33.3	7	25	578/0
	Positive	22	66.7	21	75	
**Grade**	1	9	25	11	37.9	371/0
	2	17	47.2	9	31	
	3	10	27.8	9	31	
**Type Tumor** ** : ** **IDC**	Negative	15	34.9	4	12.1	032/0
	Positive	28	65.1	29	87.9	
**Type Tumor** ** : ** **ILC**	Negative	39	90.7	30	90.9	1
	Positive	4	9.3	3	9.1	
**Tumor Type** **DCIS**	Negative	24	55.8	22	66.7	356/0
	Positive	19	44.2	11	33.3	
**Tumor Type** **LCIS**	Negative	39	90.7	33	100	128/0
	Positive	4	9.3	0	0	
**LVI**	Negative	36	83.7	16	51.6	001/0
	Positive	5	12.2	15	48.4	

SLNB: sentinel lymph node biopsy; HER2: human epidermal growth factor receptor 2; ER: Estrogen Receptor; PR: Progesterone Receptor; IDC: Invasive Ductal Carcinoma; ILC: Invasive Lobular Carcinoma; DCIS: Ductal Carcinoma in situ; LCIS: Lobular Carcinoma in Situ; LVI: lymph vascular Invasion

Ki67 is a quantitative variable, and the descriptive statistics of the mean and standard deviation of this variable in accordance with the involvement of axillary sentinel lymph nodes were reported in table 2. Independent group t-test was used to compare the average Ki67 in two groups. In the table, the average Ki67 in the positive SLNB group was 20.48, which was 7.52 points lower than the average Ki67 in the negative SLNB group. With a 95% confidence level, this difference was significant (p < 0.05). 

**Table 2:** Description of Ki67, and independent t- test to compare the mean in accordance with the status of SLNB

**P **	**t-test**	**Mean** **±** **SD**		**Variable**
		**Positive SLNB**	**Negative SLNB**	
030/0	22/2	11.82 ± 20.48	18.99 ± 28.00	**Ki67**

SLNB: sentinel lymph node biopsy

The results of the binary logistic regression test were used to predict the involvement of axillary lymph nodes. Table 3 shows the accuracy of the model in correctly classifying samples.

The results of table 3 show that based on the predictor variables of the model, it is possible to accurately predict 83.7% of individuals with a negative involvement of the axillary lymph nodes. Furthermore, 93.9% of individuals with a positive involvement of the axillary lymph nodes can be accurately predicted. The overall accuracy of the model for correct classification is 88.8%.

For the overall evaluation of the regression model, the Omnibus tests were used, which yielded a chi-square value of 23.85, which was significant (P= 0.008). It can be concluded that the overall fit of the model was acceptable, and the regression model was well-suited for predicting axillary lymph node involvement (p > 0.05). The determination coefficients obtained in logistic regression showed that the range of these coefficients was from a minimum of 0.573 for the Nagelkerke R Square coefficient to a maximum of 0.770 for the Cox & Snell R Square coefficient. It can be inferred that the predictor variables of the model were able to explain 57.3% to 77% of the changes in the criterion variable of axillary lymph node involvement, indicating a suitable explanatory power of the model. 

Table 4 shows the results of the logistic regression test to identify predictive factors of axillary lymph node involvement. The results of table 4 show that among the predictor variables of the model, three variables—IDC tumor type, LVI, and Ki67—had a significant impact on axillary lymph node involvement and could have a significant effect on classifying individuals based on axillary lymph node involvement (p < 0.05). According to the results, axillary lymph node involvement can be predicted based on the scores and results of the three variables: IDC tumor type, LVI, and Ki67. The positive relationship of the two factors, IDC tumor type and LVI, with axillary lymph node involvement indicates that when the IDC tumor type and LVI are positive, there is a higher probability that the axillary lymph nodes will also be positive (with 3.88 times higher probability for IDC tumor type and 6.75 times higher probability for the LVI factor).

Also, when the Ki67 level is lower, the probability of positive axillary lymph nodes is higher (with 3.58 times higher probability). 

**Table 3:** Checking the accuracy of the regression model in classifying the sample based on the involvement of the axillary sentinel lymph nodes

**Percentage of correct classification**	**Axillary sentinel lymphnode involvement**				**Variable**	
	**Positive**		**Negative**			
	**Percent**	**Number**	**Percent**	**Number**		
**83.7**	**6.1**	**2**	**83.7**	**36**	**Negative**	**Sentinel lymphnode involvment**
**93.9**	**93.9**	**31**	**16.3**	**7**	**Positive**	
**88.8**	**100**	**33**	**100**	**43**	**Total**	

**Table 4:** Logistic Regression Test Coefficients Table for Predicting Axillary Lymph Node Involvement Based on Predictor Variables

**Independent variable**	**Non-standard coefficient**	**Standard deviation**	**Wald statistic** **(Wald)**	**P-value**	**Odds ratio** **(OR)**
**Tumor size**	0.271	0.370	0.535	0.464	1.311
**HER2**	0.405	0.527	0.592	0.442	1.500
**ER - PR**	0.405	0.572	0.503	0.478	1.500
**Tumor grade**	-0.162	0.323	0.250	0.617	0.851
**Tumor type** ** : ** **IDC**	1.357	0.622	4.759	0.029	3.884
**Tumor type** ** : ** **ILC**	-0.025	0.801	0.001	0.975	0.975
**Tumor type** ** : ** **DCIS**	-0.460	0.480	0.915	0.339	0.632
**Tumor type** ** : ** **LCIS**	-21.036	20096.48	0.001	0.999	0.001
**LVI**	1.910	0.597	10.215	0.001	6.750
**Ki67**	-0.031	0.015	4.352	0.037	0.279

HER2: human epidermal growth factor receptor 2; ER: Estrogen Receptor; PR: Progesterone Receptor; IDC: Invasive Ductal Carcinoma; ILC: Invasive Lobular Carcinoma; DCIS: Ductal Carcinoma in situ; LCIS: Lobular Carcinoma in Situ; LVI: lymph vascular Invasion

## Discussion

In this study, we aimed to investigate the predictive factors that affect sentinel lymph node metastasis and assess the effect of histopathologic factors on sentinel lymph node metastasis. Previous studies have examined the positive predictive factors for sentinel lymph node metastasis. Out of the 470 patients who underwent surgery at the Cancer Center of Shahid Beheshti University of Medical Sciences between 2018 and 2021, 112 patients underwent sentinel lymph node biopsy. Data for 76 patients were fully recorded, of whom 43 had negative sentinel lymph nodes, and 33 had positive sentinel lymph nodes. We studied the effect of tumor grade, tumor size, hormone receptors (ER, PR, HER2), pathological tumor type (IDC, ILC, DCIS, LCIS), and LVI on sentinel lymph node metastasis. Among the factors considered, IDC and LVI were found to increase the likelihood of positive sentinel lymph node metastasis.

Several studies have demonstrated that tumor size is a significant factor in sentinel lymph node metastasis (13, 14). Based on the classification of TMN, we considered the size of the tumor to be below 2 cm, between 5 and above 5 cm. In our patients, there was no tumor size below 1 cm. Due to the lack of routine screening program, most of the patients were in larger and more palpable sizes which are detected by touching. In the mentioned studies (14), the tumor size below 5 mm and in Takada et al.’s study (15) the tumor size below 1 cm reduces the risk of involvement of the sentinel lymph nodes. However, in our study, no significant difference was seen between the size below 2 and above 2 cm. Chen's study (16) also found that grade did not have an effect on sentinel lymph node metastasis. Mao's study (17) identified pathological grade and triple-negative tumors as independent predictive factors for sentinel lymph node metastasis. The power of hormone receptors in predicting the status of sentinel lymph nodes is controversial. In some studies (18, 19), no correlation between estrogen and progesterone receptors and the involvement of sentinel lymph nodes has been seen .Gann et al.’s study (20) result that hormone receptor negative tumors have lower risk for metastasis to the sentinel lymph nodes. In Viale's et al.’s study (14), there was no correlation between (ER, ER, HER2) and sentinel lymph nodes metastasis, similarly in our study, no significant relationship was found between hormone receptors (ER/PR/HER2) and sentinel lymph node metastasis.

PVI (perivascular invasion) in Viale's et al.’s study (14) is mentioned as the strongest predictor of sentinel lymph node involvement. Furthermore, Mao's study (17) reported that angiolymphatic invasion was a strong predictor of axillary lymph node involvement. In our study, tumor lymphovascular involvement significantly increases the likelihood of sentinel lymph node metastasis. Tumor type has not consistently been reported as a significant factor in pre-sentinel lymph node metastasis in previous studies. However, Zhang's study (21) reported a higher percentage of sentinel lymph node positivity in patients with invasive ductal carcinoma. Our study also found a higher percentage of patients with positive sentinel lymph nodes in the ductal carcinoma group. Several studies have identified higher Ki67 as an effective factor in sentinel lymph node metastasis (22, 23). However, in our study, Ki67 levels were lower in the positive sentinel lymph node group, which is similar to Koyoma's study (24) where the level of Ki67 was significantly lower in the positive sentinel lymph node group as our result.

In conclusion, IDC, LVI, and lower levels of Ki67 are related to positive sentinel lymph nodes. Limitations of our study include the small number of patients and small sample size, as well as missing data. Further studies with a larger patient population are necessary to confirm the predictive factors for pre-sentinel lymph node metastasis. In our study, none of the pathological factors could 100% predict the risk of sentinel lymph node metastasis, that's why SLNB is still used as the main method of axillary lymph node evaluation.
